# Real time medical learning using the WhatsApp cellular network: a cross sectional study following the experience of a division’s medical officers in the Israel Defense Forces

**DOI:** 10.1186/s40696-016-0022-7

**Published:** 2016-08-09

**Authors:** Ofer Blumenfeld, Ronen Brand

**Affiliations:** Medical Corps, Israel Defense Forces, Tel-Aviv, Israel

**Keywords:** Medical learning, Cellular network, WhatsApp, Primary care, Mobile phone, Military medicine

## Abstract

**Background:**

Primary care medical officers (MOs) are expected to maintain self-education while serving in their units in order to maintain professional standards. With the rise of smartphone use in the Israel Defense Forces (IDF), the WhatsApp application can facilitate medical learning. To date, there has been no description of the use of this tool by MOs in a military setting. This paper aims to describe the pattern of use of a WhatsApp application group by IDF’s MOs.

**Methods:**

We collected all the information (textual and visual) uploaded to a WhatsApp group called “The Division’s Physicians”, originally established two years earlier, during a randomly selected sample month. We analyzed the pattern of its use and explored the association between the number of questions and responses uploaded to the group and the duration of service of their senders.

**Results:**

In December 2014, the “The Division’s Physicians” WhatsApp group had 41 participants. We identified 478 messages classified as questions and 531 messages classified as responses. The number of questions asked by MOs in their first 2 months of service in the battalion (median = 14.5) and the number of questions asked by MOs with more than one year of their first assignment (median = 10.5) were significantly higher than the number of questions (median = 1.0) asked by MOs in their second assignment or later (*p* values for comparisons were 0.008 and 0.012 respectively). We also found that both the number of responses provided by MOs with more than one year of service in the battalion (median = 21) and the number of responses provided by MOs in their second assignment or later (median = 5) were significantly higher than the number of responses (median = 1) provided by MOs within their first 2 months of service in the battalion (*p* value for comparisons were 0.024 and 0.039 respectively).

**Conclusion:**

We conclude from our preliminary study that a WhatsApp group can facilitate the transfer of knowledge from more experienced MOs to those with less experience.

## Background

A key element in the Israeli army’s medical effort in the field is the presence of a medical officer (MO) with full MD qualifications in every battalion, both for routine care and in the event of emergencies. An MO cares for 250–600 soldiers, depending on the type of battalion (MOs with armored and artillery corps typically treat fewer soldiers than those with infantry units). Accordingly he or she has an average of 100–450 doctor–patient encounters per month.

This MO is a recent medical school graduate whose experience to this point has been one year of hospital internship, where typical cases are very different than with a military battalion consisting primarily mainly 18–21-year-old soldiers.

The MOs begin their military service at the age of 25, at which point they receive a three-month basic military medical course before being stationed with either an infantry, armored, engineering or artillery battalion. After 2 years of service with their battalion, the MOs are promoted either to brigade MOs or to an administrative position at a central headquarters. MOs are expected to continue self-education while serving with their military units in order to maintain professional standards. Being far from formal medical educational institutions combined with being in 24-h readiness to treat the soldiers in their units prevents these MOs from keeping up-to-date with the constant changes in medical services, professional orders and even medical guidelines. The role of brigade MOs is primarily administrative. As their formal training is not superior to that of battalion MOs, they are not officially recognized as advisors.

WhatsApp is a text and picture transferring application that can assist teaching caregivers, since it enables all users to view written and visual content in real time and to respond. Johnson and colleagues showed that this application is safe and efficient for transferring orders and for peer consultations in a surgical team in a London hospital [1]. A WhatsApp group named “The Division’s Physicians” was established by a family physician in 2012.

This paper will describe the pattern of use of this WhatsApp application group.

## Methods

### Study population and sampling

Our study population included the 41 MOs who were members of this group at the time of the study. We sampled all of the messages uploaded by all of the MOs to the WhatsApp group (textual or visual) during the randomly selected month of December 2014. All messages during the sampled month were analyzed, regardless of the sender, time and day of delivery.

### Data retrieval and classification

The senior author retrieved all messages from the WhatsApp group uploaded during December 2014 and classified each message as either a question or a response. Messages were also characterized by subject, as well as by visual versus textual content. We retrieved demographic variables of the sender and his or her professional and army service experience by addressing each of the group’s participants. During the sampled month, there were three distinct subgroups of MOs based on duration of service: the first had served for up to 2 months in the battalion, the second for more than one year, and the third was serving in their second or more senior assignments.

### Study hypotheses

We had two null hypotheses: that there would be no difference between the number of questions uploaded to the group and the duration of service of their sender, and that there was no difference between the number of responses uploaded to the group and the duration of service of their sender.

### Data analysis

Analysis was done using SPSS version 21 (SPSS INC, Chicago IL). We used the Kalmagorov–Smirnof test to assess normality of variable distribution. Medians and interquartile ranges (IQRs) were used to describe the number of questions and responses for each duration of service category. We used Kruskal–Wallis non-parametric tests to explore the association between the number of questions uploaded to the group and the duration of service (as categorized to the three aforementioned groups). Post hoc analysis was performed by using Mann–Whitney test. The level of statistical significance was set to *p* = .05.

## Results

In December 2014, the “The Division’s Physicians” WhatsApp group included 41 participants: 10 MOs were in their first 2 months in the brigade (24 %), 10 MOs were on their first assignment, but with more than one year of army service (24 %), and the remaining 21 MOs were on their second or more senior assignment. This last group included three specialists (a family physician, a cardiologist and an internist). Their primary role was to observe traffic and in certain circumstances to make useful remarks. Although their response rate was negligible, they were included in the study in order to reflect the real situation. The entire group of MOs consisted of 4 females and 37 males. The 3 specialists were between the ages of 35–40 and the others were all between 25 and 30 years old.

Our analysis of the data running through the WhatsApp group identified 478 messages classified as questions and 531 messages classified as responses. 33 MOs out of 41 uploaded questions to the group, while 32 MOs provided responses.

**Fig. 1:**
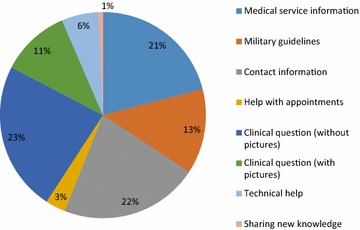
Message categories. All 1009 messages uploaded to “The Division’s Physicians” WhatsApp group categorized by eight distinctive subjects: Medical service information, Military guidelines, Contact information, Help with appointments, Clinical questions (with and without pictures), Technical help and sharing new knowledge, All figures are presented in percentages

We found seven primary message subjects: 164 (34 %) messages were clinical questions (consulting about a clinical case), 104 (22 %) messages pertained to contact information of specialists or medical services, 100 (21 %) messages concerned other information about medical services (e.g. the opening hours of an institute or hospital), 64 (13 %) messages pertained to military medicine guidelines and orders (e.g. number of sick leave days an emergency physician can recommend for a patient), 28 (6 %) messages were requests for technical help (checking lab results for a soldier while the MO is in the field treating him), 15 (3 %) messages concerned appointments for medical services and consultations, and 3 (<1 %) messages concerned sharing information about new services or guidelines. 52 clinical questions (32 % of clinical questions) contained pictures as part of the message. Data regarding message subjects presented in Fig. 1.

**Fig. 2:**
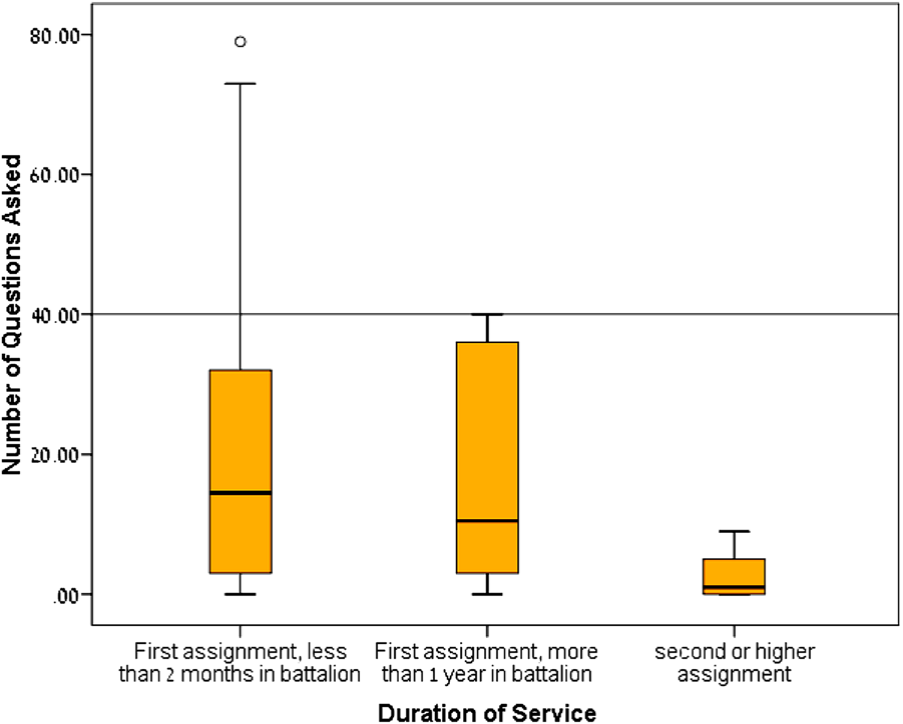
Number of questions asked based on length of service. *Box plot* showing 75th percentile, 25th percentile, median, maximum and minimum values. Outlier value is shown as a *circle*

We found that the number of questions asked by MOs in their first 2 months of service in the battalion (median = 14.5) and the number of questions asked by MOs with more than one year of their first assignment (median = 10.5) were significantly higher than the number of questions (median = 1.0) asked by MOs in their second or senior assignment (*p* values for comparisons were 0.008 and 0.012 respectively). Distribution of the number of questions by duration of service is shown in Fig. 2.

**Fig. 3:**
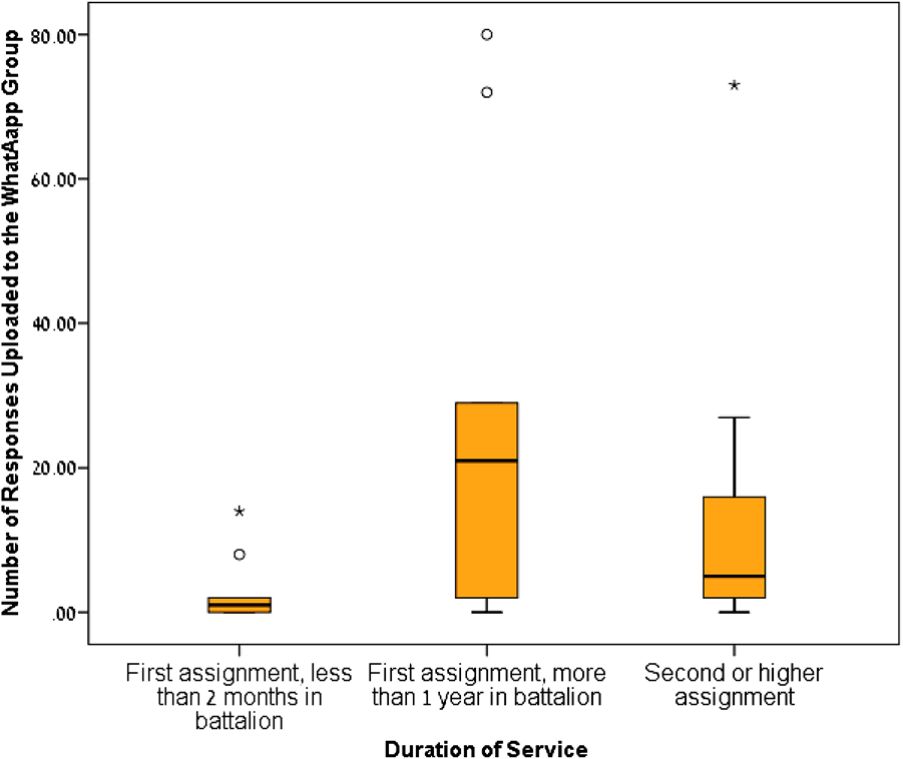
Number of responses shared to the WhatsApp group based on length of service. *Box plot* showing 75th percentile, 25th percentile, median, maximum and minimum values. Outliers and extreme values are shown as *circles* and *stars* respectively

Furthermore, both the number of responses provided by MOs with more than one year of service in the battalion (median = 21) and the number of responses provided by MOs on their second or more senior assignment (median = 5) were significantly higher than the number of responses (median = 1) provided by MOs within their first 2 months of service (*p* value for comparisons were 0.024 and 0.039 respectively). No differences were found between MOs in their second or more senior assignment and MOs with more than one year of service in the battalion in regard to the number of responses. Distribution of the number of responses by duration of service is described in Fig. 3.

## Discussion

The MO in the Israel Defense Forces Medical Corps is required to administer updated care to his patients, with minimum needs for referral to other medical practitioners. Lack of medical experience and geographical isolation of the MO dictate creative measures for continuous learning and real-time professional advice.

The battalion MO’s feeling of both professional and social isolation is common to many primary medical practitioners who work in rural clinics and in countries with large distances between cities. In 2012, the Medical Corps made a strategic decision to station a specialist in family medicine in all standard divisions in order to support and further teach new MOs serving in their first battalion assignment. This significantly improved the ability of these MOs to deal with primary medicine issues. However, the issue of bringing administrative and medical knowledge to all MOs in real-time in order to create a consistent level of education remained unresolved.

The primary method of communication remained face to face meetings between the specialists and non-specialists, and telephone consultations. With the emergence of the internet and the growing use of social media via smartphones, many attempts have been made to overcome the hardships described. In Australia, Barnett and colleagues demonstrated that a community that shared knowledge via the internet could help primary physicians to overcome the feeling of isolation and to improve communication among colleagues via chatting and sharing images and pictures [2]. In a survey conducted in the United States, McGouan et al. [3] found that 61 % of physicians used social media for finding medical information and sharing it with colleagues. Most of those physicians acknowledged that the information obtained through social media was up-to-date and of high quality. The use of Facebook in a CME program amongst pediatricians in Ohio contributed to high attendance and an increase in the use of social media as a professional tool even after 6 months of program engagement [4].

In Israel, the Rambam network served as a source of professional information for primary medical practitioners. Dikla et al., who described the use of that network, demonstrated its dual role: social, in that it belonged to the community, and professional, through the transfer of medical knowledge and expanding the formal scope of information sources available to primary physicians when making decisions [5].

The era of smart mobile phones brings a new and powerful tool for immediate online information sharing, including text, pictures and videos. Those can rapidly reach colleagues and consultant specialists. The WhatsApp mobile phone application was especially designed for this type of communication, and its use has been described in emergency rooms and other settings [5, 6].

Recently, in the US army, social media based professional military education was offered as a means to enhance learning in military academy [7].

In our study we showed that MOs in the IDF’s medical corps used the WhatsApp application for peer-advice and family practice specialist consultations. There was a statistically significant association between the MOs’ army service experience and the number of questions and responses shared in the group. MOs within their first 2 months of service in the battalion used the application primarily for asking questions, while those with more than one year of service provided responses. This facilitated the sharing of knowledge from more experienced MOs to their less experienced colleagues, which allowed better treatment with less need for referrals. It also facilitated group learning, in which all group participants were able to learn from the questions and responses posed by their colleagues. Learning subjects were diverse, including dermatology (mostly using pictures of skin lesions and rashes), various clinical fields of primary care, military primary care guidelines and orders as well as dealing with military bureaucracy. It is interesting to note that clinical issues composed only one-third of the total number of messages. This fact may suggest that the main challenge MOs in battalions face while providing medical services are of an administrative/bureaucratic nature. This hypothesis may need further investigation in future studies.

As opposed to medical care provided in rural settings in Israel, in the military, primary care is given during unconventional hours and often in a field setting. In these circumstances, there is importance great need for receiving on-the-spot advice from peers and/or specialists.

WhatsApp allows users to immediately share textual and visual content within a group of participants. This raises issues of confidentiality as well as ethical and operational security issues that need to be addressed as technology progresses [8]. For example, MOs who share clinical pictures must omit the patients’ faces in order to avoid recognition. They also must receive the patients’ consent to share the images.

There are some limitations to this study. The first concerns the lack of representation of MOs who have served more than 2 months and less than one year. A second is the one-month duration of our sampling might not represent the entire communication of the group. Third is the small number of MOs in the test group. Future research may sample similar WhatsApp groups several times during a year or for a longer period of time, in order to gain more comprehensive results. Adding more MOs from additional WhatsApp groups could help us better understand these learning processes.

Further research is needed in order to evaluate the impact of WhatsApp in military medicine and its influence on primary care physicians’ knowledge, self-confidence and decision making.

## Conclusion

We can conclude from our preliminary study that a WhatsApp group can facilitate knowledge transfer from experienced MOs to less experienced ones. Thus, it should be further evaluated for its place as a tool for medical learning in the military setting.

## Abbreviations

MO: Military Officer
IDF: Israel Defense Forces

## References

[1] Johnston MJ, King D, Arora S, Behar N, Athanasiou T, Sevdalis N, Darzi A (2015). Smartphones let surgeons know WhatsApp: an analysis of communication in emergency surgical teams. Am J Surg.

[2] Barnett S, Jones SC, Caton T, Iverson D, Bennett S, Robinson L (2014). Implementing a virtual community of practice for family physician training: a mixed-methods case study. J Med Internet Res..

[3] McGowan BS, Wasko M, Vartabedian BS, Miller RS, Freiherr DD, Abdolrasulnia MJ (2012). Understanding the factors that influence the adoption and meaningful use of social media by physicians to share medical information. J Med Internet Res..

[4] Klein M, Niebuhr V, D’Alessandro D (2013). Innovative online faculty development utilizing the power of social media. Acad Pediatr.

[5] Cohen DA, Levy M, Cohen Castel O, Karkabi K (2013). The influence of a professional physician network on clinical decision making. Patient Educ Couns.

[6] Srivastava PV, Sudhan P, Khurana D, Bhatia R, Kaul S, Sylaja N, Moonis M, Pandian JD. Telestroke a viable option to improve stroke care in India. Int J Stroke. 2014;9 Suppl A100:133–4. 10.1111/ijs.12326 25042038

[7] Kimball RA, Byerly JM (2013). To make Army PME distance learning work, make it social. Military Review..

[8] Moe TA. Social Media and the U.S. Army: Maintaining a Balance. http://oai.dtic.mil/oai/oai?verb=getRecord&metadataPrefix=html&identifier=ADA544887. Accessed 28 June 2016.

